# The Benefits of Utilizing Total Body Composition as a Predictor of Cardiorespiratory Fitness Based on Age: A Pilot Study

**DOI:** 10.3390/ijerph19095758

**Published:** 2022-05-09

**Authors:** Jeffery L. Heileson, Zacharias Papadakis, Ahmed Ismaeel, Kathleen A. Richardson, Ricardo Torres, LesLee Funderburk, Andrew Gallucci, Panagiotis Koutakis, Jeffrey S. Forsse

**Affiliations:** 1 Department of Health Human Performance and Recreation, Baylor University, Waco, TX 76706, USA; jeffery_heileson@baylor.edu (J.L.H.); katie_adair1@baylor.edu (K.A.R.); ricardo_torres1@baylor.edu (R.T.); andrew_gallucci@baylor.edu (A.G.); 2 Department of Sport and Exercise Sciences, Barry University, Miami Shores, FL 33161, USA; zpapadakis@barry.edu; 3 Clinical Muscle Biology Laboratory, Biology Department, Baylor University, Waco, TX 76798, USA; ais247@uky.edu; 4 Department of Physiology, University of Kentucky, 780 Rose St., MS508, Lexington, KY 40536, USA; 5 Human Sciences and Design, Baylor University, Waco, TX 76798, USA; leslee_funderburk@baylor.edu

**Keywords:** total body composition, cardiorespiratory fitness, percent body fat, bone mineral composition

## Abstract

**Simple Summary:**

In this paper, we demonstrate the importance of assessing body composition as a whole and the usefulness in predicting cardiopulmonary fitness as an indicator of overall health. Therefore, the significance of this manuscript is to improve clinicians’ and researchers’ understanding of the importance of evaluating total body composition when assessing overall health and the serious implications that body composition has on an individual’s health as they age.

**Abstract:**

Maximal oxygen consumption (VO_2max_) has been associated with body fat percentage (%BF) or fat free mass. However, most analyses do not consider total body composition (TBC) as defined by %BF, fat free mass index (FFMI—a height-adjusted measure of muscle mass), visceral adipose tissue, and bone mineral content (BMC). The aim of this study was to determine if TBC predicts cardiorespiratory fitness in healthy adults and if a relationship exists in young and older adults. Sixty healthy individuals (age group 1 (AG1, ≤35 years), *n* = 35; age group 2 (AG2, >35 years), *n* = 25) were screened in a cross-sectional study and retrospectively examined. All participants completed a full body DEXA scan and a standardized multistage treadmill test to determine VO_2_max. A multiple linear regression analysis was performed to examine the relationship between TBC and VO_2max_. The multiple regression model showed an overall significant effect for TBC (*p* < 0.001, R^2^ = 0.282). When analyzed by age group, the regression model of TBC was not significant in young adults (AG1, *p* = 0.319, R^2^ = 0.141), but significant in older adults (AG2, *p* < 0.001, R^2^ = 0.683). Significant predictors of VO_2max_ in the older cohort were %BF (*β* = −0.748, *p* = 0.001) and BMC (*β* = 0.014, *p* = 0.002). Total body composition predicted VO_2max_ in a small cohort of healthy adults. This study highlights the importance of TBC for cardiovascular health, especially in mid-to later-life individuals.

## 1. Introduction

Maximal oxygen consumption (VO_2max_) is an essential component to measure cardiorespiratory fitness and athletic performance [1,2,3]. VO_2max_ can also be used as a valuable clinical tool to identify individuals at risk for chronic illnesses, primarily cardiovascular disease (CVD) [4]. Individuals with lower cardiorespiratory fitness are five times more likely to experience all-cause mortality associated with CVD than individuals with higher cardiorespiratory fitness [5]. Currently, VO_2max_ remains one of the most well-established standards for assessing overall health in healthy and diseased populations, and is quantified as a MET level (1 metabolic equivalent = 3.5 mL/kg/min) and is a method used to establish exercise intensity levels [4,6].

Another significant contributor to increased mortality arising from CVD is obesity, which accounts for a three-fold increase in the risk of CVD mortality in obese individuals compared to healthy-weight individuals [7,8]. Obesity is widely determined using body mass index (BMI) (kg/m^2^) [9]. However, total body composition (TBC) is not taken into account with BMI; therefore, discrepancies persist in evaluating individual health. TBC is separated into three main categories of assessment: percent body fat (%BF), lean body mass (LBM), and bone mineral content (BMC) [10]. In addition, the introduction of the capabilities to assess visceral adipose tissue (VAT) has gained increased interest over the last 10 years. Due to the vast clinical implications that VAT has on multiple populations—since VAT has been linked with a heightened risk of CVD when compared to subcutaneous %BF—VAT needs to be an additive marker of TBC [11]. 

While VO_2max_ has been associated with %BF or LBM, most analyses do not consider TBC, VAT, and BMC [12,13]. Previous research utilized and compared either %BF, LBM, or BMC to VO_2max_ individually without using TBC [14]. Moreover, the majority of prior research studies focus on specific participant populations (e.g., young athletic males and females, middle-aged males, older females) and do not account for the overall general population (e.g., age, sex) [15,16]. Therefore, the aim of this study was to ascertain if TBC (%BF, LBM (kg), BMC (g), and VAT (g)) predicts cardiorespiratory fitness in generally healthy adults and whether this relationship exists in young and older adults. We hypothesized that TBC would have a significant influence in predicting VO_2max_ and that there would be a significant difference in TBC between young vs. older adults. 

## 2. Materials and Methods

The University Institutional Review Board approved the research study with human subjects (#1574622-3). To ensure the safety of all participants, the ethical guidelines of the 1975 Declaration of Helsinki were employed by all research personnel in accordance with the institution’s human research committee. Individuals who qualified to participate in the study were first presented with a written informed consent document regarding the specifications of the study. Research personnel then verbally explained all procedures thoroughly and instructed the participants to ask questions if they had any. Participants signed and returned the informed consent document and underwent an extensive health history and exercise questionnaire before being admitted into the study (see further participant details below).

### 2.1. Subjects

A total of sixty individuals were enrolled to participate in the study. Participants (*n* = 45 men; *n* = 15 women) were healthy between the ages of 20 to 60 years of age and were physically active based on an exercise questionnaire, which met exercise recommendations established by the American College of Sports Medicine: achieving the minimum exercise recommendations of 150 min of moderate-intensity exercise, or 75 min of high-intensity exercise on a weekly basis over the last 3 months [17,18]. Participants had to be non-obese, non-smokers, having never been diagnosed with cardiovascular or metabolic diseases and were currently not taking any prescription medications except vitamins. Participants were required to visit their primary care physician on an annual basis to ensure the absence of medical diagnoses. Each participant provided a health history questionnaire before being admitted into the study. Participants were excluded if they were diagnosed with any form of CVD, diabetes, dyslipidemia, cancer, or any other health disparity. The recruitment of participants resulted in 87 individuals interested in participating in the study; of 87, 20 were excluded due to the previous diagnoses of cardiometabolic diseases, 5 were excluded due to fitness level, and 2 were excluded due to time conflicts. Research subjects’ baseline demographics are provided in Table 1.

### 2.2. Study Procedure

All participants reported to the research lab after completing a modicum of a four-hour fast from caloric-containing substances. All participants were encouraged to consume water during this time. Participants were told to abstain from exercise for a minimum of 24 h prior to arriving at the lab to undergo body composition and VO_2max_ testing. Participants were asked to wear shorts, a t-shirt, and a pair of comfortable jogging/running shoes. All the experimental procedures were reviewed again with each participant, and any questions they had concerning the study were answered.

### 2.3. Total Body Composition Assessment

Baseline height and weight were determined using an electronic scale and stadiometer (Seca 703), participants removed their shoes prior to stepping on the scale. Prior to the body composition assessment, participants were asked to void their bladder and remove all forms of metal, which would influence BMC. Total body composition was measured (e.g., lean, fat, bone tissue) via dual-energy X-ray absorptiometry (DXA, Discovery DXA™, Hologic^®^, Bedford, MA, USA). Participants were placed according to the National Health and Nutrition Examination Survey (NHANES) recommendations: body supine, head straight, a small amount of space between the arms and torso, hands fixed on the table, and feet together [11,19]. To establish body regions, the regions of interests’ analysis lines were placed as portrayed in the NHANES Body Composition Procedures Manual: horizontal lines were placed lower to the skull and at the height of the iliac crest; vertical lines were established and aligned adjacent to the vertebral column and between the legs; diagonal lines were for both glenohumeral joints and femoral necks [19]. To promote and maintain consistency of scan analyses, two researchers analyzed the entire study of dual-energy X-ray absorptiometry (DXA) (Hologic Inc., Bedford, MA, USA) scans by utilizing the Hologic APEX software (version 4.6).

### 2.4. Baseline Assessment

Resting heart rate was recorded using a Polar belt (H7™, Polar Wearlink^®^ Lake Success, New York, NY, USA), which was positioned on the sternum. Resting blood pressure was recorded in the seated position after the participant had rested for 5 min. Blood pressure measurements were obtained using a manual standing sphygmomanometer and stethoscope. Cuff size was selected based on the size of the participant’s arm. The participant’s waist circumference was taken using a manual measuring tape (the average of three measurements was used). All measurements throughout the test were performed by trained exercise physiologists.

### 2.5. Exercise Testing

All participants performed a maximal aerobic exercise treadmill test to determine the VO_2max_ and maximal heart rate, allowing for cardiorespiratory fitness classification. Respiratory gases (VO_2_ and VCO_2_) were measured using a facemask connected to an integrated respiratory gas analysis system (TrueOne 2400™, ParvoMedics^®^, Sandy, UT, USA). Respiratory gases and heart rate were monitored throughout the test. One-minute averages were recorded at rest and throughout the test in order to accurately compare the exercise values to baseline. The exercise test began after one minute of standing to obtain resting respiratory values. The starting treadmill speed was determined using the participant’s 5K race pace. Speed was increased by 1 mph every 3 min for the first three stages. The incline was set at 0% for the first three stages and was increased by 2% increments each additional minute with the speed remaining constant at stage three, until the end of the test criteria was reached [20]. To achieve a true VO_2max_, individuals had to obtain a respiratory exchange ratio (RER) of >1.1, a plateau of VO_2_, and/or achieve an 85% or higher age-predicted max heart rate [21]. The test was terminated if the research team observed signs or symptoms that warranted test termination or at the participant’s request. The exercise protocol used was based on previous studies that utilized treadmill running to achieve maximal cardiorespiratory fitness. The majority of our subject population were runners, cyclists, cross-fit, or multi-sport trained. Therefore, a running protocol was more suitable for these individuals.

### 2.6. Statistical Analyses

Means and standard deviations were determined for all clinical and body composition measurements. Normality was assessed via visual inspection of the histograms and P-P plots and the independence of residuals was determined by the Durbin–Watson statistic. Baseline characteristics and body composition measures were compared based on age groups using *t*-tests. Associations between the TBC variables and VO_2max_ were reported as Pearson correlation coefficients (*r*). Multiple linear regression analysis was used to test the relationship between VO_2max_ and TBC (%BF, LBM, VAT, and BMC). The regression analysis was conducted based on groups dichotomized by age group (AG1: ≤ 35 vs. AG2: > 35). Age groups were determined based on the mean age of the cohort. Data were analyzed with SPSS version 27 (IBM SPSS, Chicago, IL, USA). Significance was set *a priori* at *p* < 0.05.

## 3. Results

### 3.1. Participants

Table 1 shows the physical characteristics of participants in AG1 and AG2. Briefly, weight, height, BMI, and VO_2max_ are similar between age groups; however, older participants exhibited a significantly lower resting heart rate. The body composition variables for all participants and between age groups are reported in Table 2. Weight, fat mass, %BF, LBM, BMC, and VAT were similar between age groups (*p* > 0.05).

### 3.2. Correlations between VO_2max_ and Total Body Composition Variables

Based on univariate analysis, two of the predictors, %BF (*r* = −0.488, *p* < 0.001) and BMC (*r* = 0.300, *p* = 0.020), were significantly correlated with VO_2max_, while VAT (*r* = −0.061, *p* = 0.642) and LBM (*r* = 0.238, *p* = 0.067) were not. For participants in AG1, %BF (*r* = −0.322, *p* = 0.059), LBM (*r* = −0.055, *p* = 0.755), VAT (*r* = −0.209, *p* = 0.227), and BMC (*r* = −0.152, *p* = 0.384) were not correlated with VO_2max_. However, in AG2, %BF (*r* = −0.617, *p* = 0.001), LBM (*r* = −0.557, *p* = 0.004), and BMC (*r* = 0.651, *p* < 0.001) were significantly correlated with VO_2max_, while VAT (*r* = 0.151, *p* = 0.470) was not correlated with aerobic capacity.

### 3.3. Multiple Linear Regression Models

For the whole cohort (*n* = 60), TBC significantly predicted VO_2max_ (R^2^ = 0.282, *p* < 0.001). Body fat percentage was the best independent predictor of VO_2max_ (*p* = 0.001, 95% CI: −0.931, −0.238)). While VAT, BMC, and LBM contributed to the model, they were not significantly associated with aerobic fitness. For AG1, the regression model was not significant (*p* = 0.319). For AG2, the regression model was significant (R^2^ = 0.683, *p* < 0.001), with %BF and BMC being the strongest predictors of VO_2max_. Results of the multiple regression model for AG2 are presented in Table 3. In participants older than 35, the linear regression model demonstrated that for every 1% increase in %BF, VO_2max_ decreased by 0.748 mL/kg/min, and for every 10 g increase in BMC, VO_2max_ increased by 0.14 mL/kg/min.

## 4. Discussion

In our study, TBC was a significant predictor of cardiorespiratory fitness in young and middle-aged healthy adults with the %BF exhibiting the strongest, albeit moderate, correlation with VO_2max_. However, this relationship was much stronger in the middle-aged group, indicating the importance of body composition influence on cardiorespiratory fitness during aging. In AG2, %BF, BMC, and LBM were strongly correlated with VO_2max_, whereas in AG1, there were no significant correlations between any measure of body composition and aerobic fitness.

Aging uniquely influences many physiological functions, and the most observable are those regarding body composition changes; namely, the progressive loss of bone and muscle mass and the accumulation of body fat (i.e., visceral, subcutaneous) [22]. Similarly, cardiorespiratory fitness tends to decline with age in men and women [23]. While we demonstrated that TBC is predictive of VO_2max_ in healthy adults without cardiometabolic disease, the predictive power of TBC was much greater in middle-aged adults, accounting for 68.3% of the variance in VO_2max_. Given that TBC and VO_2max_ encompass the integrated physiological functions of the cardiovascular, respiratory, metabolic, and biomechanical systems and are associated with similar health outcomes, it should be no surprise that there is a close relationship between TBC and cardiorespiratory fitness, especially in an aging population.

Improved quality of life through the maintenance of a healthy %BF is consistently the focus of researchers and healthcare professionals [24,25]. An increase in adipose tissue and a decrease in LBM is routinely observed in older individuals. Furthermore, it is directly associated with a rise in age-related pathologies, influencing the decline in functional capabilities [26]. By assessing %BF and categorizing the type (VAT vs. subcutaneous) of adipose tissue, we believe a more comprehensive health appraisal can be achieved when predicting cardiorespiratory fitness. Our study assessed both total %BF and VAT as part of the TBC model that we employed to predict VO_2max_. Independently, only %BF was significantly correlated with VO_2max_ (*p* < 0.001). The results indicate that cardiorespiratory fitness decreased by 0.585 mL/kg/min for every 1% increase in %BF. These outcomes are indicative of the current observations of changes in %BF with age in the general population [27]. In general, the average older adult loses 3–8% LBM every decade, starting at 30 years of age [28,29]. Middle-aged adults also gain a total of 0.5 to 1 kg per year, with the majority of weight gain arising from an increase in %BF [30]. These observed changes in weight gain are believed to be linked more to lifestyle than typical aging factors [8]. To account for the wide variance of our cohort’s age, we separated participants into two groups, AG1 vs. AG2 (>35). Similar to the total cohort results, %BF significantly predicted VO_2max_ in the AG2 group (*p* < 0.001); however, this was not observed in the AG1 group (see Table 3). These results indicate that VO_2max_ declines by 0.748 mL/kg/min for every 1% increase in total %BF in middle-aged adults. This indicates a clinical significance (average of 2 Mets lower) between VO_2max_ and %BF in populations as they age, which we believe is of greater importance when compared to using only statistical significance. Our results further strengthen the need to prioritize maintaining a healthy %BF throughout the aging process to decrease the development of various health disparities. By maintaining a healthy %BF, the risk of developing CVD, health disparities, and early mortality, decreases. In our study, no significant effects were observed with sex differences in relation to %BF and VAT on cardiorespiratory fitness. Though assessing VAT remains a critical factor in determining health risk appraisal, in this study, when using %BF and VAT, only the total %BF accurately predicted or exhibited a significant correlation with VO_2max_.

The importance of BMC throughout the aging process has been well documented [22]. BMC is highly integrated into standard health risk assessments for aging individuals and influences many health outcomes related to longevity, quality of life, risk of bone fracture, and mortality [31]. Exercise, such as resistance training and high-impact sports, significantly improves and maintains BMC in numerous healthy and diseased populations [32,33,34]. In our study, total BMC was used to predict VO_2max_ independently and as a part of the TBC model. BMC was assessed on the whole cohort and by group (AG1 vs. AG2). Collectively, BMC was not a significant predictor of cardiorespiratory fitness; however, there was a difference between groups. In the AG2 group, BMC significantly predicted VO_2max_ (*p* < 0.02), while BMC did not predict VO_2max_ in the AG1 group. In addition, the correlation between BMC and VO_2max_ was positive, indicating that as BMC increases, VO_2max_ increases by 0.14 mL/kg/min^−1^ for every 10 g increase in BMC. In previous studies, VO_2max_ was used to predict BMC and bone mineral density in healthy, athletic, and obese individuals and was determined to be positively correlated [14,15]. These results appear to contradict our AG1 group’s results when applied in a reverse manner (VO_2_ predicting BMC vs. BMC predicting VO_2_). Since the participants in these studies were in a similar age range (18–35 years) to the younger cohort in our study, these results appear to contradict the results found within the AG1 group of our study. However, there is a difference in population characteristics between studies, limiting the comparison of results. While these previously included individuals who were professional athletes, healthy, or obese, our cohort consisted of healthy, physically active individuals with normal BMIs. Further, beyond the younger group, our study included individuals 20–60 years of age. Therefore, our results further support the additive health benefits of maintaining BMC throughout the aging process to attenuate the development of comorbidities or increased early mortality.

Limitations of the current study include (1) potential sex differences due to an unequal number of males and females, (2) relying on participants to be honest to abstain from exercise and nutrition during the required time frame, (3) participants presenting accurate health records and information to be admitted into the study, (4) not assessing older individuals over the age of sixty-one, (5) not assessing baseline blood values for cardiometabolic diseases, and (6) due to the nature of the study being a pilot study. Additionally, we did not monitor the participants’ diet or exercise in the days leading up to the health assessment.

## 5. Conclusions

In conclusion, TBC, identified as LBM, %BF, VAT, and BMC, predicts VO_2max_ in a modest cohort of healthy adults between the ages of 20 to 60 years without cardiometabolic diseases. However, there was a strong relationship between TBC and VO_2max_ in participants older than 35 years, with the strongest predictors being BMC and %BF. Thus, this study highlights the importance of body composition for cardiovascular health, especially in mid-to later-life individuals. Continued research into the implementation of healthy lifestyle habits to promote healthy TBC is needed to ensure the best quality of life is obtained during the aging process.

## Figures and Tables

**Table 1:** Participant characteristics by age group (AG).

	AG1 (≤35)	AG2 (>35)	*p*-Value
Sample Size (M/F)	35 (29/6)	25 (16/9)	---
Age	27.7 (4.2)	46.9 (7.2)	---
Weight (kg)	78.5 (15.4)	75.0 (13.1)	0.353
Height (m)	1.8 (0.1)	1.7 (0.1)	0.398
BMI (kg/m^2^)	25.1 (3.8)	25.2 (3.7)	0.975
Resting Heart Rate (bpm)	73.2 (10.2)	64.8 (9.8)	0.005
VO_2max_ (mL/kg/min)	45.4 (7.3)	41.5 (8.0)	0.053

Data are mean (SD).

**Table 2:** Body Composition.

	≤35 (*n* = 35)	>35 (*n* = 25)	*p*-Value
Weight (kg)	78.5 (15.4)	75.0 (13.1)	0.353
FM (kg)	14.7 (6.4)	16.2 (5.7)	0.345
BF (%)	19.2 (5.7)	21.4 (7.0)	0.171
LBM (kg)	61.2 (12.2)	55.8 (10.7)	0.078
BMC (kg)	2.7 (0.3)	2.6 (5.4)	0.416
VAT (kg)	0.3 (0.1)	0.3 (0.1)	0.834

Abbreviations: body fat percentage, %BF; bone mineral content, BMC; fat mass, FM; lean body mass, LBM; visceral adipose tissue, VAT. Data are mean (SD).

**Table 3:** Multiple linear regression analysis model for AG2 (>35).

VO_2max_ (mL/kg/min)				
	*β*	SE	*t*-Value	*p*
%BF	−0.748	0.189	−3.961	0.001
VAT	0.014	0.012	1.196	0.246
BMC	0.014	0.004	3.635	0.002
LBM	0.000	0.000	−1.985	0.061
TBC	44.72	9.020	4.958	<0.001

Abbreviations: AG2, age group 2 (>35); %BF, body fat percentage; VAT, visceral adipose tissue; BMC, bone mineral content; LBM, lean body mass; TBC, total body composition (composite of %BF, VAT, BMC, LBM). Statistical significance set at *p* < 0.05 (bold).

## Data Availability

Not applicable.
